# Aversive gustatory learning and perception in honey bees

**DOI:** 10.1038/s41598-018-19715-1

**Published:** 2018-01-22

**Authors:** Marie Guiraud, Lucie Hotier, Martin Giurfa, María Gabriela de Brito Sanchez

**Affiliations:** 1 Centre de Recherches sur la Cognition Animale (CRCA), Centre de Biologie Intégrative (CBI), Université de Toulouse; CNRS, UPS, 31062 Toulouse cedex 9, France; 2 Present Address: Queen Mary University of London, School of Biological and Chemical Sciences, Biological and Experimental Psychology, Mile End Road, London, E1 4NS United Kingdom

## Abstract

Taste perception allows discriminating edible from non-edible items and is crucial for survival. In the honey bee, the gustatory sense has remained largely unexplored, as tastants have been traditionally used as reinforcements rather than as stimuli to be learned and discriminated. Here we provide the first characterization of antennal gustatory perception in this insect using a novel conditioning protocol in which tastants are dissociated from their traditional food-reinforcement role to be learned as predictors of punishment. We found that bees have a limited gustatory repertoire via their antennae: they discriminate between broad gustatory modalities but not within modalities, and are unable to differentiate bitter substances from water. Coupling gustatory conditioning with blockade of aminergic pathways in the bee brain revealed that these pathways are not restricted to encode reinforcements but may also encode conditioned stimuli. Our results reveal unknown aspects of honey bee gustation, and bring new elements for comparative analyses of gustatory perception in animals.

## Introduction

Taste is the sense that distinguishes between chemical compounds and the sensations they produce based on contact with chemoreceptors^1^. It allows discriminating edible from non-edible items and is, therefore, crucial for survival. Research on taste perception has revealed both common and specific mechanisms of gustatory processing between the few species that have been extensively investigated until now^2^. A proper understanding of taste perception across species thus requires expanding the spectrum of species studied and a careful consideration of their feeding biology as a determinant of taste processing mechanisms. Unlike many other animals, insects present taste receptors, which are not restricted to the region around the mouth but which may be found on the antennae, legs, wings and oviposition organs, among others. In addition, whereas in vertebrates taste receptors are stimulated by chemicals in solution, insects have the capacity to perceive chemicals on dry surfaces^3^. It has been thus suggested that in the case of insects, the sense of taste should be rather termed “gustation” or “contact chemoreception”^4^.

Among insects, the honey bee *Apis mellifera* offers a unique opportunity to study gustation because of the relevance of this sense in its foraging activities and social communication^1^. Yet, despite having a well-established model status for research on olfaction^5–7^ and vision^8–10^, the gustatory perception of this insect has remained largely unexplored^1,2^. Behavioral studies on honey bee gustation have been restricted to an *appetitive* framework because the distinctive hallmark used to assess gustation is the spontaneous proboscis extension response (PER), an appetitive reflex elicited by the contact of sucrose and other sweet tastants with the antennae, tarsi or mouth parts^11,12^. Aversive substances do not elicit PER so that their perception can only be assessed indirectly, via inhibition of PER following sucrose stimulation^13^, or by mixing them with sucrose solution^14^, which generates the problem of gustatory masking by sucrose^15^. PER has been intensively used to study olfaction as harnessed bees can be easily conditioned to associate an odorant (the conditioned stimulus or CS) with a reward of sucrose solution (the unconditioned stimulus or US) delivered to their antennae and mouth parts^16^. Bees that learn the odor-sucrose association extend the proboscis to the odorant that predicts reinforcement. Owing to this CS role of odors, it has been possible to study olfactory generalization, discrimination and memorization, and to determine the principles and mechanisms of olfactory perception^6,17,18^. A similar success has not been possible in the case of gustation because sucrose and other tastants are persistently used as reinforcements (i.e. as US) rather than as stimuli to be learned. This limitation has hindered analyses on gustatory learning and discrimination in bees and calls for novel conditioning procedures in which tastants act as stimuli predicting reinforcement (i.e. as CS), instead of being mere reinforcements.

Here we introduce three novel achievements: 1) we established a new learning paradigm in which bees learn tastants delivered to their antennae as CS and in which reinforcement is not gustatory. We took advantage of the sting extension reflex (SER), which can be elicited in harnessed honeybees by a mild electric shock^19–21^, and paired the delivery of tastants with shock in order to induce a gustatory conditioning of SER. We trained bees under a differential conditioning regime, in which they had to learn to distinguish a punished tastant from an unpunished tastant. Differential conditioning was used as it improves discrimination in various species and sensory modalities and reveals a real inability to distinguish between stimuli in the case of unsuccessful learning^22–31^. 2) Using this protocol, we characterized antennal gustatory perception and learning in bees. We studied if appetitive substances that are innately preferred acquire an aversive value through their association with shock and if bees discriminate between tastants of the same or different quality. 3) Finally, we explored the neural underpinnings of gustatory learning by focusing on aminergic pathways in the bee brain. We show that these pathways not only encode reinforcement signals^32–39^, but may also signal the presence of gustatory conditioned stimuli.

## Results

### Honey bees learn gustatory aversive associations and acquire defensive responses to innately preferred food

**Figure 1:**
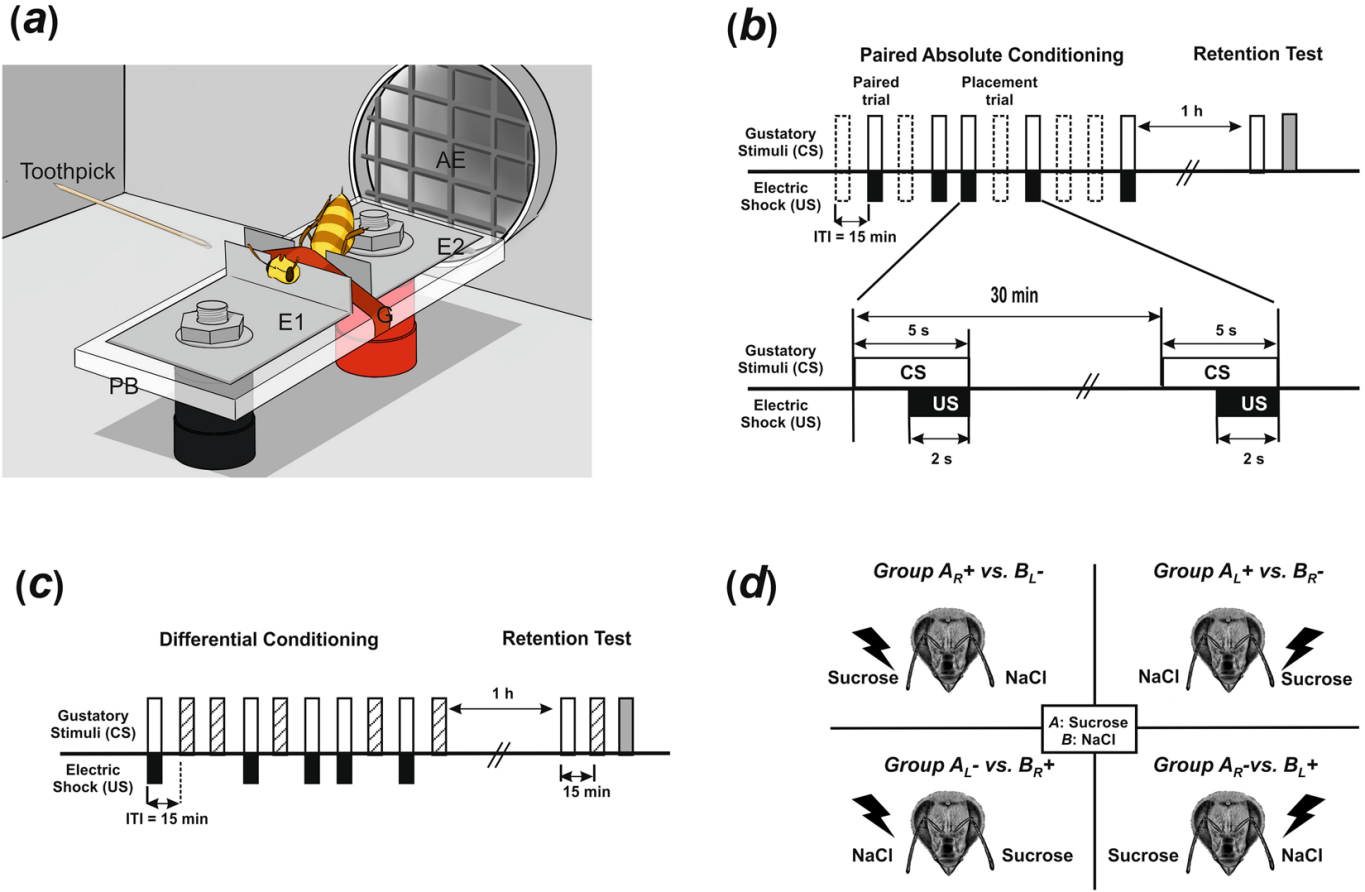
Associative gustatory conditioning of the sting extension reflex (SER) in honeybees. (**a**) View of a honeybee in the experimental set-up (drawing by Marie Guiraud). The bee is fixed between two brass plates (E1, E2) set on a Plexiglas basis (PB) by a girdle (G) that clamped the thorax to restrain mobility. EEG cream is smeared on the two notches to ensure good contact between the metal plates and the bee. The bee closes a circuit and receives a mild electric shock (7.5 V) which induces the sting extension reflex (SER). A tastant is delivered by a toothpick contacting the antennae. Pheromone contamination is avoided via an air extractor (AE) which is continuously on and placed behind the bee. (**b**) Experimental schedule for absolute gustatory conditioning of SER. Bees were trained with five paired presentations of a single tastant, sucrose (white bars), and electric shock (black bars). Five blank trials (dashed bas) were interspersed between conditioning trials to allow comparison with an unpaired group (not shown). The intertrial interval (ITI) was 15 min. One hour after the end of conditioning a retention test was performed in which the CS was delivered without shock (white bar). At the end of the test, the shock was delivered alone (grey bar) to check the integrity of SER. Each trial consisted of a gustatory stimulation (conditioned stimulus or CS) that lasted 5 s, followed by an electric shock (unconditioned stimulus or US) that started 3 s after tastant onset and lasted 2 s. (**c**) Experimental schedule for differential gustatory conditioning of SER. Bees were trained to discriminate two tastants (CS), one (white bars) paired 5 times with shock delivery (black bars, US) and the other presented 5 times without shock punishment (dashed bars). The intertrial interval (ITI) was 15 min. One hour after the end of conditioning a retention test was performed in which the two CSs were delivered without shock (white and dashed bars). At the end of the test, the shock was delivered alone (grey bar) to check the integrity of SER. Each punished trial was identical to those described in (**a**). Non-punished trials were similar except that no shock was delivered. (**d**) Experimental groups trained under the differential-conditioning regime to achieve balance between antennal sides and reinforcement contingencies of both tastants (bee head adapted from picture by Cyril Fresillon, @CNRS).

To determine if SER can be conditioned using gustatory stimuli delivered to the antennae as CS, we trained harnessed bees (Fig. 1a) under an absolute-conditioning regime, pairing five presentations of 1 M sucrose solution with five stimulations with a mild electric shock of 7.5 V (Fig. 1b). Bees had thus to learn to extend defensively the sting to sucrose, an appetitive stimulus that never triggers this reaction. Bees in an unpaired group received the same number of gustatory and shock stimulations but in a non-contingent manner.

**Figure 2:**
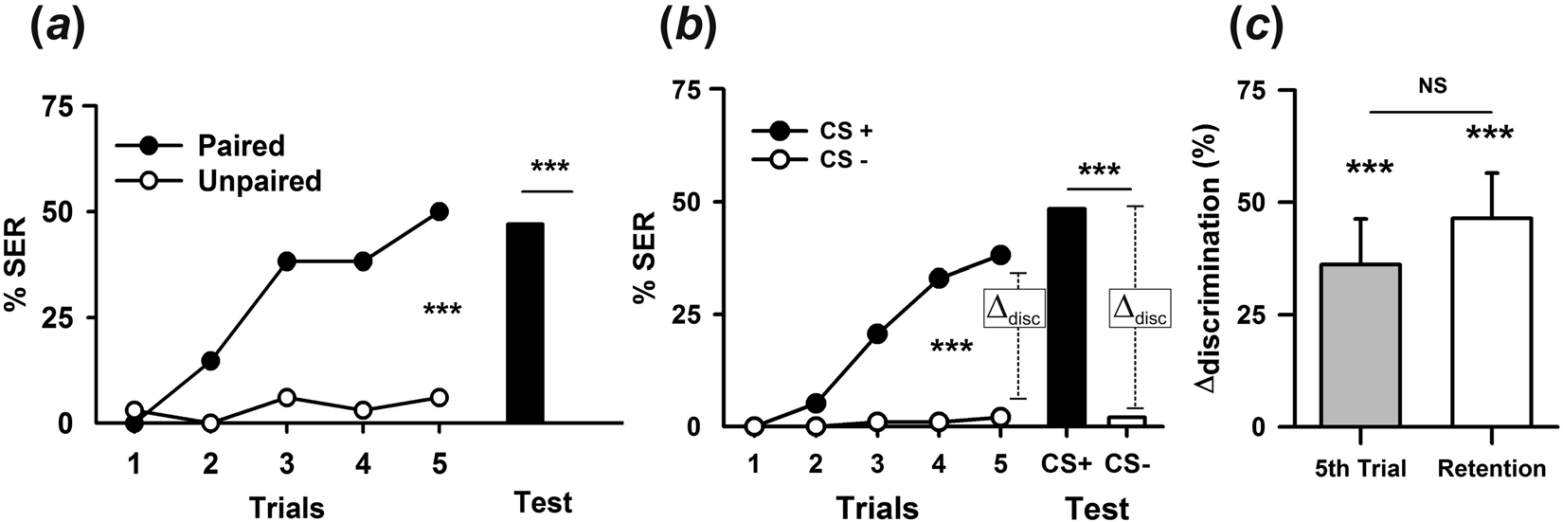
Absolute and differential gustatory conditioning of the sting extension reflex (SER). (**a**) Bees trained under absolute conditioning to associate a preferred tastant, 1 M sucrose solution, with a 7.5 V electric shock. Conditioned responses (% SER) of a ‘paired group’ of bees having experienced five pairings of sucrose and electric shock (black circles; *n* = 34) and of an ‘unpaired group’ having experienced non-contingent sucrose and electric shock stimulations (white circles; *n* = 33). Only bees of the paired group learned the association and extended their sting in response to sucrose. One hour after conditioning, a gustatory aversive memory was present in the paired (black bar) but not in the unpaired (white bar, zero % in this case) group. ***P < 0.0001. (**b**) Differential conditioning of 1 M sucrose vs 3 M NaCl. Conditioned responses (% SER) of a group of bees (*n* = 97) subjected to five CS+ and five CS− trials. Bees learned the gustatory discrimination and responded significantly more with a SER to the punished tastant than to the non-punished one at the end of training. One hour after conditioning, they remembered the learned associations (bars). Discrimination levels (Δ_disc_ = SER_CS+_ − SER_CS−_) reached in the last conditioning trial and in the retention test are shown. (**c**) Discrimination levels (Δ_disc_) reached at the end of differential conditioning and in subsequent retention tests. Both Δ_disc_ were significantly different from zero and did not differ from each other. NS: non-significant; ***P < 0.0001. Error bars on Δ_disc_ levels represent 95% confidence intervals.

Bees of the paired group significantly increased their defensive response to sucrose (Fig. 2a; ANOVA for repeated measurements: *F*_1,66_ = 35.00; *P* < 0.0001) while bees of the unpaired group did not change their sucrose responsiveness (*F*_1,65_ = 0.24; *P* = 0.63). Thus, the increase in SER observed in the paired group was due to associative learning and not to the simple experience with sucrose and shock. The performance of both groups differed significantly (*F*_1,65_ = 40.00; *P* < 0.0001) and the evolution of responses during trials was also different between groups, as shown by a significant interaction (*F*_4,260_ = 5.82; *P* < 0.002). One hour after the last conditioning trial, bees of the paired group remembered the aversive tastant and exhibited defensive SER to it, while bees of the unpaired did not respond with SER to sucrose (Fig. 2a, bars; Mc Nemar test; *χ*^2^ = 31.03; *P* < 0.0001). A robust aversive gustatory memory for sucrose was therefore established in the paired group, even if this tastant is originally an innate appetitive stimulus for honey bees.

As sucrose elicits spontaneous proboscis extension responses (PER), we quantified the occurrence of this appetitive unconditioned behavior in both the paired and the unpaired group. Typically, the % of PER in a group of motivated foragers varies between 90% and 100%. We aimed at determining if learning of a sucrose-shock association degrades the appetitive value of sucrose, thus diminishing spontaneous PER to it. Supplementary Fig. S1 shows that even if PER to sucrose never reached typical levels, it remained high (between 55% and 60%) and constant along trials in the unpaired group (*F*_4,128_ = 0.15; *P* = 0.96). In the paired group, levels of PER (between 40% and 60%) were also lower than typical response levels and did not vary along trials (*F*_4,132_ = 1.12; *P* = 0.35). Note that in this group, sucrose was contingent with shock so that PER responses occurred in parallel with SER responses shown in Fig. 2a. Comparing both groups did not reveal significant differences between them (*F*_1,65_ = 2.03; *P* = 0.16). One hour after conditioning, PER to sucrose was lower in the paired group (44%) compared to that of the unpaired group (64%) but the difference was again not significant (*F*_1,65_ = 2.59; *P* = 0.11). Thus, despite the formation of an aversive memory associating sucrose and electric shock, the intrinsic appetitive value of sucrose was highly but not integrally preserved.

### Honey bees learn to discriminate different gustatory stimuli delivered to separate antennae

We then studied gustatory discrimination by separating gustatory inputs at the level of the two antennae and associating to them different reinforcements. We trained bees under a differential-conditioning regime (Fig. 1c) to reveal the actual capacity of bees to distinguish between gustatory stimuli as this training improves discrimination and reduces generalization^22–31^. Bees were trained to discriminate 1 M sucrose from 3 M NaCl, two solutions with strong appetitive and aversive reinforcing properties^40^, respectively. One tastant was paired with shock (CS+) and the other not (CS−). Bees had to learn to extend the sting only to the punished tastant. Four groups were conditioned to balance the contingencies between gustatory stimulus, antennal side and reinforcement (Fig. 1d).

Antennal side was not relevant as shown by the performances of the bees conditioned with sucrose+ vs. NaCl− (left vs. right: *F*_1,36_ = 0.31; *P* = 0.58) and with sucrose− vs. NaCl+ (left vs. right: *F*_1,35_ = 0.54; P = 0.47). Thus, within each discrimination, data were pooled. We then determined if there were asymmetries in tastant learning depending on which tastant was paired with shock. Both groups learned the gustatory discrimination (sucrose+ vs. NaCl− : *F*_1,36_ = 42.80; *P* < 0.001; sucrose− vs. NaCl+ : *F*_1,35_ = 113.00; *P* < 0.001) and there were no detectable differences between them (*F*_1,73_ = 1.10; *P* = 0.30). The results were thus pooled and shown as a CS+ vs. CS− discrimination (Fig. 2b).

Taste discrimination was highly significant (Fig. 2b; *F*_1,73_ = 142.7; *P* < 0.001) and evolved rapidly during the five conditioning trials (*F*_4,292_ = 23.56, *P* < 0.001). The interaction was also significant (*F*_4,292_ = 16.80; *P* < 0.0001), showing that bees increased SER to the punished tastant and did not respond to the unpunished tastant. Post-hoc Tukey tests showed that gustatory discrimination occurred from the 2^nd^ trial on (*P* < 0.0001). The discrimination level reached in the last conditioning trial (Δ_discrimination_ = SER_CS+_ − SER_CS−_; dashed vertical line in Fig. 2b), which is a measure of learning efficiency and will be used henceforth, was significantly different from zero and confirmed discrimination between both tastants (Fig. 2c: grey bar; one-sampled t test: *t*_96_ = 7.05; *P* < 0.0001).

Gustatory learning induced a memory that was retrieved 1 h later, when bees were presented with each tastant on its corresponding antennal side and without shock (Fig. 2b, bars; *χ*² = 25.29, *P* < 0.001). In this case, the Δ_discrimination_ (Fig. 2c: white bar) was significantly different from zero (*t*_96_ = 9.12; *P* < 0.0001) and similar to that observed at the end of conditioning (t_96_ = 1.64; *P* = 0.11).

**Figure 3:**
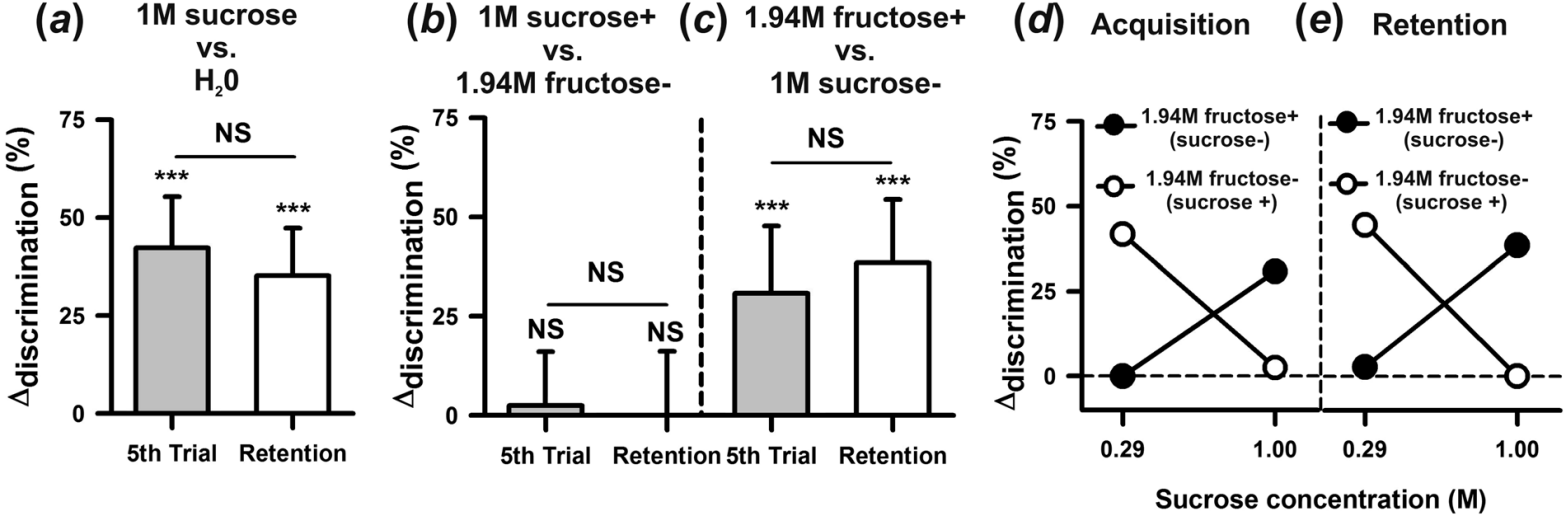
Sweetener perception and discrimination in honeybees. (**a**) Discrimination learning of 1 M sucrose vs. distilled water. Δ_discrimination_ values reached in the last conditioning trial and in the retention test. Bees (*n* = 71) learned the discrimination and remembered it 1 h later. (**b**) Discrimination learning between sweeteners, 1 M sucrose (+) and 1.94 M fructose (−). Δ_discrimination_ values reached in the last conditioning trial and in the retention test when 1 M was sucrose punished (+) and 1.94 M fructose was unpunished (−) (*n* = 40). Bees, which prefer the former to the latter, were unable to learn the difference between these two sweet tastants and therefore showed no retention 1 h after conditioning. (**c**) Discrimination learning between 1.94 M fructose (+) and 1 M sucrose (−). Δ_discrimination_ values reached in the last conditioning trial and in the retention test after differential conditioning when 1.94 M fructose was punished (+) and 1 M sucrose was unpunished (*n* = 39). In this case, bees learned efficiently the difference between the two sweet solutions and remembered it 1 h later. (**d**) Asymmetry in sweetener discrimination learning. Δ_discrimination_ values in the last conditioning trial as a function of variable sucrose concentration. The concentration of fructose solution was constant (1.94 M). Values for 1 M sucrose correspond to those reported in (**b**) and (**c**) (gray bars). When sucrose concentration was devaluated so that fructose became more attractive, the asymmetry in sweetener discrimination was reversed. (**e**) Asymmetry in sweetener retention. Same as in (**d**) but for retention performances. NS: non-significant; ***P < 0.0001. Error bars on Δ_disc_ levels represent 95% confidence intervals.

Because each tastant was delivered on the same antenna throughout the conditioning procedure, we performed a control experiment to determine if bees learned the association of antennal side with shock rather than the gustatory discrimination itself (supplementary Fig. S2). We delivered the same tastant (1 M sucrose) alternately to each antenna, one antenna being reinforced and the other not. The Δ_discrimination_ indexes obtained for the last conditioning trial and for the retention test were not different from zero (Fig. 3d; *t*_63_ = 0.50; *P* = 0.62 and *t*_63_ = 0.77; *P* = 0.44, respectively), thus showing that bees were unable to learn the discrimination between sucrose punished and sucrose non-punished based on antennal side.

Taken together, the results of this section show that bees learn and memorize efficiently a discrimination between gustatory conditioned stimuli of different modalities (sweet, salty). Importantly, the side of punishment was not learned *per* se and no stimulus-specific asymmetries in learning were detected.

### Discrimination between sweeteners depends on their hedonic rank

We next focused on discrimination between sweeteners and studied if bees can distinguish them using their antennae. As a positive control, we determined the capacity of bees to distinguish concentrated 1 M sucrose solution from distilled water (Fig. 3a). There were no differences in discrimination learning according to antennal side both when water (left vs. right: *F*_1,33_ = 0.07; *P* = 0.78) and sucrose (left vs. right: *F*_1,34_ = 0.005; *P* = 0.82) were reinforced. Similarly, there were no differences according to which tastant was reinforced (*F*_1,69_ = 0.01; *P* = 0.91) so that results could be pooled. Bees learned efficiently the discrimination between sucrose solution and distilled water and remembered it 1 h later, thus reaching significant Δ_discrimination_ indexes both at the end of conditioning (Fig. 3a; gray bar; *t*_70_ = 6.45; *P* < 0.0001) and in the retention test (Fig. 3a; white bar; *t*_70_ = 5.82; *P* < 0.0001).

We then studied the capacity of bees to discriminate two sweeteners with different hedonic values based on their antennae. For honey bees, sugars with different nutritional values differ in their attractiveness and are distinguished and ranked accordingly (fructose < glucose < sucrose)^41,42^. In experiments performed in our laboratory, we could also show that upon antennal stimulation with these sugars, all at a concentration of 30% (w/w), responses to sugar significantly increase from fructose to sucrose, thus reproducing the same ranking^43^. We conditioned bees to discriminate solutions of 1 M sucrose and 1.94 M fructose (both corresponding to a 35% w/w solution), knowing that foragers prefer sucrose over fructose when both have similar w/w concentrations^41,43^. There were no significant differences according to antennal side both when sucrose (*F*_1,38_ = 0.013, *P* = 0.91) and fructose (*F*_1,40_ = 0.75; *P* = 0.39) were reinforced. However, performance varied significantly depending on which sweetener was paired with shock (*F*_4,308_ = 18.12, *P* < 0.001) so that results of both discriminations are presented separately (Fig. 3b: Δ_discrimination_ for sucrose+ vs. fructose−; Fig. 3c: Δ_discrimination_ for sucrose− vs fructose+ ). When sucrose was paired with shock (Fig. 3b), bees were unable to learn the discrimination between sucrose and fructose (*t*_39_ = 0.37; *P* = 0.71) and, therefore, did not show significant retention 1 h later (*t*_39_ = 0; *P* = 1.0). However, when fructose was paired with shock (Fig. 3c), bees learned efficiently the discrimination between fructose and sucrose (*t*_38_ = 3.69; *P* < 0.001) and remembered it 1 h later (*t*_38_ = 4.87; *P* < 0.0001). This asymmetry suggests that accentuating hedonic differences by associating a less preferred sweetener with an aversive outcome facilitates discrimination; on the contrary, blurring these differences by associating a preferred sugar with an aversive outcome renders discrimination impossible.

**Figure 4:**
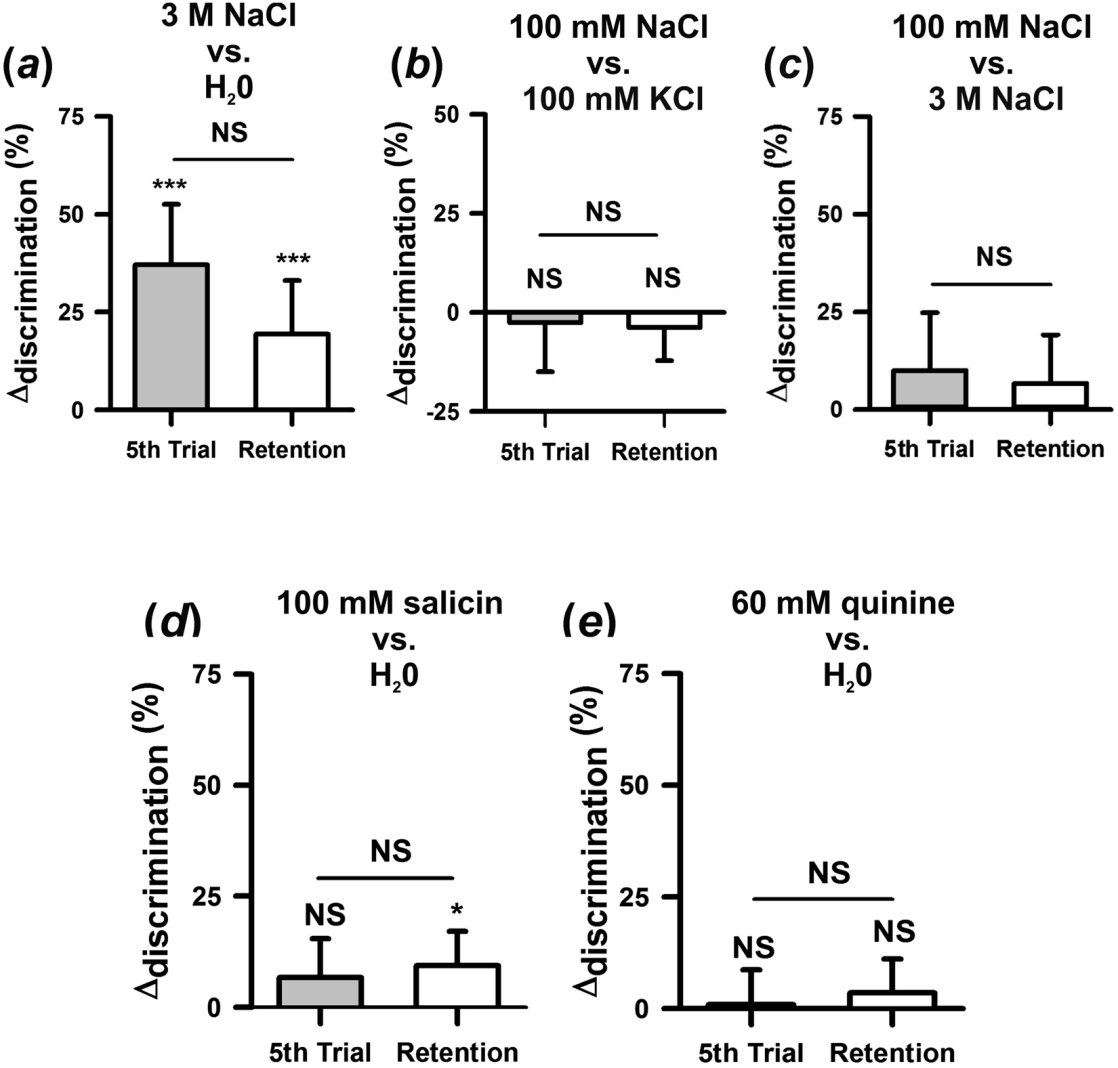
Salty and bitter antennal perception and discrimination. (**a**) Antennal discrimination learning of 3 M NaCl vs. distilled water. Δ_disc_ values reached in the last conditioning trial and in the retention test. Bees (*n* = 62) learned to distinguish the salty solution from water and remembered the discrimination 1 h later. (**b**) Antennal discrimination learning between two salty tastants. Δ_disc_ values reached in the last conditioning trial and in the retention test after differential conditioning of 100 mM NaCl vs. 100 mM KCl (*n* = 79). Bees were unable to learn the difference between these two salty tastants and therefore showed no retention 1 h after conditioning. (**c**) Antennal discrimination learning between two concentrations of the same salty tastant. Δ_disc_ values reached in the last conditioning trial and in the retention test after differential conditioning of 100 mM NaCl vs. 3 M NaCl (*n* = 60). Bees were unable to learn the difference between these two concentrations of the same tastant using their antennae and therefore showed no retention 1 h after conditioning. (**d**) Antennal discrimination learning of 100 mM salicin vs. distilled water. Δ_disc_ values reached in the last conditioning trial and in the retention test. Bees (*n* = 75) were unable to learn the discrimination between the bitter substance and water; differentiation in the test, although significant, reached only 9%. (**e**) Antennal discrimination learning of 60 mM quinine vs. distilled water. Δ_disc_ values reached in the last conditioning trial and in the retention test. Bees (*n* = 112) neither learned nor retained the discrimination between the bitter substance and water. NS: non-significant; *P < 0.05; **P < 0.01, ***P < 0.0001. Error bars on Δ_disc_ levels represent 95% confidence intervals.

We tested this hypothesis by devaluating the sucrose solution with respect to the fructose solution. Bees were trained to discriminate the same 1.94 M fructose solution from a 0.29 M sucrose solution, i.e. 3.5 times lower than that of the previous experiment. Again, there were no significant differences according to antennal side both when sucrose (*F*_1,34_ = 0.25, *P* = 0.62) and fructose (*F*_1,34_ = 0.74; *P* = 0.79) were reinforced but performance varied significantly depending on which sugar was paired with shock (*F*_4,280_ = 22.624, *P* < 0.0001). Compared to the previous experiment, the opposite results were found: when sucrose 0.29 M (now the poorer solution) was paired with shock, bees learned the discrimination (Fig. 3d*, left white circle*: *t*_35_ = 5, *P* < 0.0001). By contrast, when fructose 1.94 M (now the richer solution) was paired with shock, bees could not learn the discrimination (Fig. 4d, *left black circle*: *t*_35_ = 0, *P* = 1.0). Figure 3d shows the inversion of Δ_discrimination_ values in the 5^th^ conditioning trial between this experiment (left circles; sucrose 0.29 M) and the previous one in which a higher concentration of sucrose was used (right circles; sucrose 1 M). A similar result was obtained for retention performances (Fig. 3e). Taken together these results confirm that when sweeteners differ in their hedonic value, enhancing differences by pairing a non-preferred sweetener with an aversive stimulus facilitates discrimination; on the contrary, associating the preferred sweetener with an aversive stimulus suppresses discrimination.

### Honeybees exhibit limited antennal discrimination within the salty modality

We next studied antennal discrimination within the salty modality. We first determined if bees learn to discriminate a concentrated salty solution of 3 M NaCl from distilled water using their antennae. There were no differences in discrimination learning according to antennal side both when water (*F*_1,28_ = 0.71; *P* = 0.41) and NaCl (*F*_1,28_ = 1.83; *P* = 0.86) were reinforced. Similarly, there were no differences according to which tastant was reinforced (*F*_1,60_ = 0.44; *P* = 0.51). Results were thus pooled and showed as a CS+ vs CS− discrimination. Figure 4a shows that bees learned efficiently this control discrimination (*t*_61_ = 4.81; *P* < 0.0001) and remembered it 1 h later (*t*_61_ = 2.83; *P* < 0.01).

We then assessed the antennal capacity to discriminate two different saline solutions with the same concentration. Preferences for specific saline solutions and concentrations have only been reported for water foragers^44^, a group of bees that was not used in our experiments. We chose nevertheless two saline solutions in the range of reported preferences (100 mM NaCl and 100 mM KCl)^44^ and asked if bees learn to discriminate them. There were no significant differences according to antennal side both when NaCl (*F*_1,38_ = 0.65, *P* = 0.42) and KCl (*F*_1,37_ = 0.01, *P* = 0.91) were reinforced. Also, no asymmetry in discrimination was found between both saline solutions (*F*_1,77_ = 1.98, *P* = 0.16). The pooled performance (Fig. 4b) showed that bees were unable to learn the difference between NaCl and KCl (*t*_78_ = 0.41; *P* = 0.69) and thus exhibited no retention 1 h later (*t*_78_ = 0.90; *P* = 0.37).

We finally studied the capacity to distinguish two concentrations of the same saline solution using their antennae. We trained bees to discriminate 100 mM from 3 M NaCl to determine if asymmetries between these two tastants would arise as in the case of sucrose solutions differing in concentration. There were again no differences according to antennal side both when the diluted (F_1,28_ = 0, NS) and the concentrated (F_1,28_ = 0.09, NS) NaCl solutions were punished. Despite the difference between both NaCl concentrations, no learning asymmetries were detected (F_1,58_ = 0.21, NS). The pooled performance (Fig. 4c) shows that bees did not learn the difference between both concentrations of NaCl (*t*_59_ = 1.35; *P* = 0.18) and therefore exhibited no retention (*t*_59_ = 1.07; *P* = 0.29). The absence of asymmetry and the incapacity to learn the difference between both NaCl concentrations using their antennae thus suggest that these solutions were ranked similarly by bees in our experimental situation.

### Honeybees are unable to distinguish bitter stimuli from water by means of their antennae

Bitter substances [*although the term ‘bitter’ refers to a human sensation and can be hardly attributed to an insect, we use it here as usually done in the insect gustatory literature*] are biologically relevant due to their potential toxicity. The capacity of bees to detect bitter substances is controversial^13,45^. We thus aimed at determining if bees distinguish concentrated bitter solutions from distilled water by means of their antennae. We first trained bees to discriminate distilled water from a concentrated solution of 100 mM salicin (Fig. 4d). There were no significant differences according to antennal side both when salicin (F_1, 36_ = 0.0003, P = 0.99) and water (F_1,35_ = 0.46, P = 0.50) were reinforced. Also, no asymmetry in discrimination was detected between both tastants (F_1,73_ = 1.79, P = 0.18) so that performances could be pooled and analyzed in terms of a CS+ vs CS− discrimination. Surprisingly, bees were unable to learn the discrimination between the concentrated salicin solution and water (Fig. 4d, t_74_ = 1.52; P = 0.13). In the retention test a small, yet significant Δ_discrimination_ was found (Fig. 4d, t_74_ = 2.41; P < 0.05) but the difference in responses between the CS+ and the CS− was only 9%. Thus, honeybees could barely differentiate the concentrated salicin solution from distilled water by means of their antennae.

We verified this finding by conditioning another group of bees to discriminate a concentrated solution of quinine (60 mM) from distilled water at the antennal level (Fig. 4e). As in the previous experiment, there were no differences according to antennal side both when quinine (*F*_1,45_ = 0.01, *P* = 0.93) and water (*F*_1,44_ = 0.11, *P* = 0.75) were reinforced. No asymmetry in discrimination was found between quinine and water (*F*_1,91_ = 0.0002, *P* = 0.99) so that performances could be pooled (Fig. 4e). As for salicin, bees were unable to learn the discrimination between the concentrated quinine solution and distilled water using their antennae (*t*_111_ = 0.23; *P* = 0.82). Therefore, they exhibited non-significant retention (*t*_111_ = 0.94; *P* = 0.35). Thus, bees could not differentiate the quinine solution from distilled water by means of their antennae despite the remarkable gustatory differences that these two tastants have for humans and the significant involvement of antennae in bee gustatory perception^1^.

### Aversive conditioning of preferred food requires convergence of octopaminergic and dopaminergic pathways

To study the neural bases of aversive gustatory learning, we coupled our conditioning protocol with pharmacological blockade of aminergic neurotransmission in the bee brain. Biogenic amines are essential neurotransmitters conveying reinforcement information in the insect brain. In honeybees, octopamine (OA) mediates the reinforcing properties of sucrose reward in olfactory appetitive learning^32–34^ while dopamine (DA) mediates the reinforcing properties of electric-shock in olfactory aversive learning^35^. Learning a sucrose-shock association would imply, therefore, that 1) the octopaminergic pathway conveys CS rather than US information, 2) the dopaminergic pathway conveys aversive US information and 3) a close connectivity should exist between these two aminergic circuits in order to favor the labeling of sucrose with an aversive value. In this scenario, blockade of DA signaling should suppress gustatory aversive learning irrespectively of the tastant conditioned, due to the impossibility of experiencing properly the electric shock used as aversive reinforcement. On the contrary, blockade of OA signaling should suppress gustatory aversive learning only if sucrose is used as conditioned stimulus.

**Figure 5:**
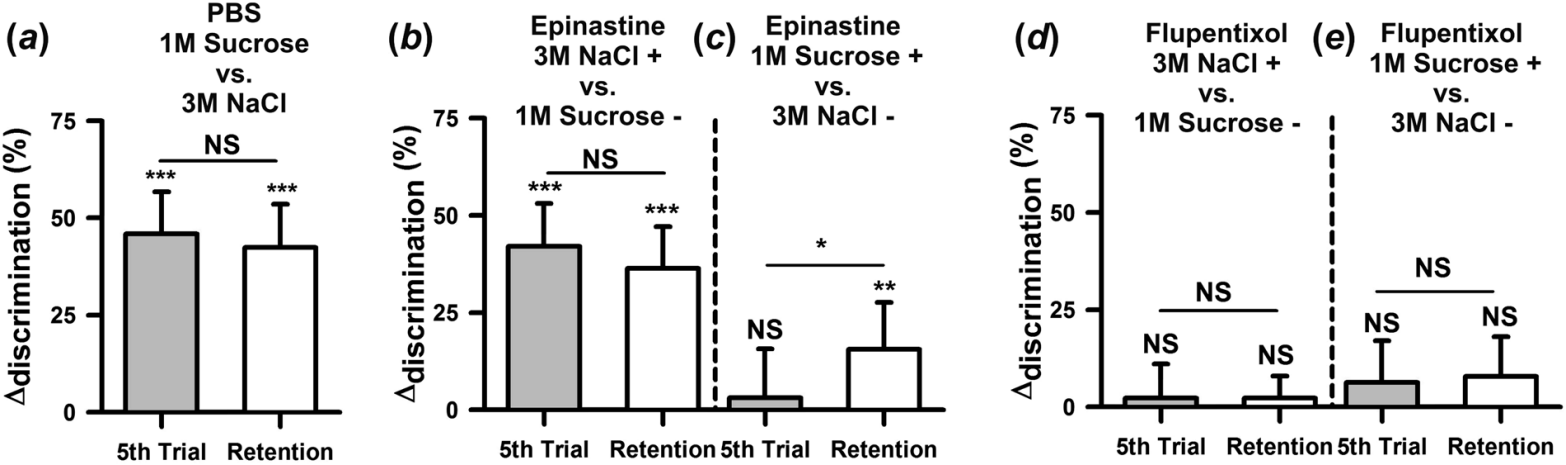
Aminergic pathways underlying gustatory SER conditioning. (**a**) Antennal discrimination learning of 1 M sucrose vs. 3 M NaCl in control bees. PBS-injected bees (*n* = 85) learned the discrimination and remembered it 1 h later. (**b**) Blockade of octopaminergic signaling for 3 M NaCl (+) vs. 1 M sucrose (−). Δ_disc_ values of epinastine-injected bees (*n* = 88) show that the discrimination was learned and retained, thus showing no effect of octopaminergic blockade. (**c**) Blockade of octopaminergic signaling for 1 M sucrose (+) vs. 3 M NaCl (−). Δ_disc_ values of epinastine-injected bees (*n* = 64) show that in this case the discrimination was not learned; retention, although significant, reached only 15%. (**d**) Blockade of dopaminergic signaling for 3 M NaCl (+) vs. 1 M sucrose (−). Flupentixol-injected bees (*n* = 86) did not learn the discrimination and therefore did not exhibit significant retention. (**e**) Blockade of dopaminergic signaling for 1 M sucrose (+) vs. 3 M NaCl (−). Flupentixol-injected bees (*n* = 64) did neither learn nor memorize the gustatory discrimination. NS: non-significant; *P < 0.05; **P < 0.01, ***P < 0.0001. Error bars on Δ_disc_ levels represent 95% confidence intervals.

To test this hypothesis, we trained bees to discriminate 1 M sucrose from 3 M NaCl. Thirty minutes before conditioning, bees were injected into the brain via the ocellar tract with PBS (control solution), *epinastine hydrochloride* (OA receptor antagonist^46^), or *cis-(Z)-flupentixol dihydrochloride* (DA receptor *antagonist*^47^). PBS-injected bees behaved as intact bees as they learned to discriminate sucrose and NaCl (Fig. 5a). There were neither significant differences according to antennal side both when sucrose (*F*_1,31_ = 2.11; *P* = 0.16) and NaCl (*F*_1,50_ = 0.26; P = 0.61) were reinforced, nor asymmetries in tastant learning depending on which tastant was paired with shock (*F*_1,83_ = 0.03; *P* = 0.87). As already shown (see Fig. 2b,c), gustatory discrimination was highly significant. Δ_discrimination_ indexes were significantly different from zero both at the end of conditioning (Fig. 5a; *t*_84_ = 8.44; *P* < 0.0001) and in the retention test (*t*_84_ = 7.50; *P* < 0.0001), thus showing that the injection procedure did not impair *per se* gustatory learning and discrimination via the antennae.

Bees injected with two different concentrations of epinastine (3.5 × 10^–4^ mM and 3.5 mM) exhibited similar performances (*F*_1,150_ = 0.16, *P* = 0.69) so that their results were pooled. No significant differences were found according to antennal side both when sucrose (*F*_1,62_ = 2.24; *P* = 0.14) and NaCl (*F*_1,86_ = 1.84; P = 0.18) were reinforced. Discrimination performances were asymmetric, depending on which tastant was associated with shock. When NaCl was punished and sucrose not, epinastine-injected bees learned the discrimination (Fig. 5b) and their Δ_discrimination_ indexes were significantly different from zero both in the last conditioning trial (*t*_87_ = 7.59; *P* < 0.0001) and in the retention test (*t*_87_ = 6.73; *P* < 0.0001). On the contrary, when sucrose was punished (Fig. 5c), epinastine-injected bees did not show any sign of discrimination during learning (*t*_63_ = 0.50; *P* = 0.62). In the retention test, the Δ_discrimination_ was significant (*t*_63_ = 2.61; *P* < 0.0001) but differentiation reached only 15%. These results indicate that the association between NaCl and shock does not engage octopaminergic signaling while that between sucrose and shock does.

Bees injected with flupentixol (1.94 × 10^–4^ mM and 1.94 mM) showed no differences according to antennal side both when sucrose (*F*_1,62_ = 1.25; *P* = 0.27) and NaCl (*F*_1,83_ = 1.95; P = 0.17) were reinforced. Irrespectively of which tastant was paired with shock, bees in which blockade of dopaminergic signaling was achieved were unable to discriminate sucrose and NaCl. Yet, Δ_discrimination_ indexes were slightly higher when sucrose was paired with shock compared with NaCl paired with shock (*F*_1,147_ = 5.43; *P* < 0.05) so that results are presented separately. When NaCl was punished and sucrose not (Fig. 5d), flupentixol-injected bees did not learn to discriminate both tastants (*t*_85_ = 0.53; *P* = 0.60) and therefore they exhibited no retention (*t*_85_ = 0.81; *P* = 0.42). Similarly, when sucrose was punished and NaCl not (Fig. 5e), flupentixol-injected bees were unable to learn the discrimination between both tastants (*t*_63_ = 1.16; *P* = 0.25) and exhibited no retention (*t*_63_ = 1.52; *P* = 0.13). These results show that irrespectively of the tastant paired with shock the injection of a dopaminergic antagonist abolishes the capacity to learn the discrimination between sucrose and NaCl, consistently with the necessity of dopaminergic neurotransmission for shock signaling.

Thus, the octopaminergic pathway, which in honey bees is thought to mediate appetitive US signaling in the case of sucrose, can also mediate CS signaling in gustatory SER conditioning. They also indicate that this pathway converges with the dopaminergic pathway in the bee brain to facilitate the learning of an association between sucrose and an electric shock.

## Discussion

Our results show that tastants delivered to the antennae, a main gustatory organ^48^, and paired with a mild electric shock, elicit the defensive sting extension response (SER), thereby revealing the aversive value acquired through learning^49^. This novel conditioning protocol allowed investigating antennal gustatory perception of bees owing to the fact that tastants acted as conditioned stimuli without confounding feeding/reinforcing components. We show that even innately preferred food such as profitable sucrose solutions could be learned as potential aversive stimuli, a result that reveals the impressive learning ability of honeybees, which can thus revert innate preferences through experience^50^.

### Discrimination between sweeteners is determined by hedonic ranks

Discrimination between sweeteners via the antennae was possible if hedonic differences were accentuated by associating a less preferred sweetener with shock and a preferred sweetener with the absence of shock. If, on the contrary sweetener differences were blurred by inversing this contingency, discrimination became impossible. This asymmetry is the consequence of enhancing or reducing the value contrast between two stimuli that need to be discriminated. A similar situation was found in fruit flies trained to discriminate either sugar solutions or mixtures of sucrose and bitter compounds^51^.

In addition, we showed that this principle does not apply to gustatory stimuli belonging to different modalities (e.g. sweet vs. salty, sweet vs. water, salty vs. water). When tastants belonged to different modalities, they were equally well learned in association with the shock so that discrimination was possible in both directions, even if they differed in their hedonic value (e.g. sucrose vs NaCl). It seems, therefore, that a key aspect to explain asymmetry in gustatory discrimination is not only the existence of hedonic preferences but also the engagement of the same or different circuits of gustatory detection and coding. When the same or similar circuits are recruited by a pair of tastants (as in the case of sucrose and fructose) the asymmetry would arise; by contrast, the use of different pathways for gustatory detection and coding would facilitate symmetric discrimination.

The same principle could apply within other gustatory modalities. For instance, discriminating low and high concentrations of saline solution could result in a similar asymmetry. Yet, how bees rank saline solutions in terms of their hedonic value is unknown. Only water foragers have been studied for their saline preferences^44^, and bees in our experiments were not water foragers. Choosing the stimuli for an experiment on asymmetric discrimination requires knowing *a priori* which of both tastants is the preferred one and which the less preferred one (as in the case of sucrose and fructose), an information that is absent for many tastants. It could be conceived that a concentrated saline solution is less preferred than a diluted one and that in such a case asymmetric discrimination could take place. Alternatively, this mechanism could be restricted to the sweet modality if the other modalities engage within-pathways that facilitate discrimination.

### The case of bitter substances: a limited gustatory repertoire

A notable particularity of the bees’ antennal gustation was the incapacity to distinguish concentrated bitter solutions from distilled water via antennal stimulation, despite the distasteful sensations induced by these substances in humans. From the five different taste qualities existing in humans - sweet, salt, sour, bitter and umami -, only two – sweet^52,53^ and salt^13,45,52–54^ have been identified beyond doubt in the honeybee. While the perception of sour and umami stimuli has not been explicitly studied in this insect, results obtained so far indicated that bees are incapable of detecting directly bitter substances and toxins via their antennae^43^. Yet, these results were obtained within an appetitive framework as they involved the use of sucrose and the quantification of proboscis extension response (PER). For instance, one antenna was first stimulated with sucrose, thereby inducing PER, and subsequently a bitter substance was delivered on the other antenna to determine if it induced proboscis retraction. Conversely, one antenna was first stimulated with the bitter substance and then the other antenna was stimulated with sucrose to determine if it had the strength to overcome the potential inhibition of PER by the bitter substance^13^. None of these experiments showed an aversive effect of the bitter substances tested^13^. Yet, as mentioned above, using an appetitive response (PER) adds confounding factors, which can be eliminated by dissociating the gustatory stimulation from a feeding response. We thus chose the sting extension reflex as the response to be conditioned as it has no feeding connotations. It allowed retesting the effect of bitter substances in a different behavioral framework, related this time to a defensive context. The fact that we found the same lack of effect, despite the difference in behavioral contexts confirms previous conclusions on a reduced antennal sensitivity for bitter substances in honey bees.

It could be argued that the high concentrations of quinine and salicin used in our work could have damaged bitter receptors on the antennae, thus explaining the observed lack of effect of these substances. Yet, our prior electrophysiological work investigating the presence of bitter receptors on these appendages^13^ used very low concentrations of quinine and salicin ranging from 0.01 mM to 1 mM and in no case, cell responses to these substances could be detected. Thus, the incapacity to discriminate bitter solutions from water reflects a reduced capacity to detect these substances at the antennal level rather than a damage of gustatory receptors by high concentrations of bitter substances. The choice of these concentrations was based on recent works with free-flying honey bees and bumble bees^55–58^, which used them as the penalty associated with non-rewarded colors to enhance color discrimination. In these works, an improvement of color discrimination was observed when quinine penalized incorrect choices^55–58^. Despite this result, and consistently with our previous findings, we found that quinine and salicin could not be discriminated from distilled water. The results on color discrimination in free-flying bees could be due to the contrast between an expected sugar reward and the sensing of a non-sugary substance. Furthermore, rejection could also be due to the malaise induced by these substances following inadvertent consumption^43,59^.

Bees have, therefore, an antennal gustatory perception that is poorer than that of other insects such as the fruit fly *Drosophila melanogaster*, which detects not only sweet^60^ and salty substances^61^ but also numerous bitter substances with its antennal gustatory receptors^62,63^. Whether bitter detection is possible via other sense organs of the honey bee remains to be determined. Bitter substances could be sensed through the inhibitory effect they exert on sucrose receptor neurons, for instance those located on the antennae^13,45^. Thus, the results of experiments in which bitter substances are mixed with sucrose solution are difficult to interpret, as rejection of these mixtures may sometimes not reflect avoidance of a distasteful stimulus but of a non-sugary substance.

### A new conditioning protocol for the study of gustatory learning and discrimination

In gustatory SER conditioning, gustatory stimuli act as conditioned stimuli (CS) and not as reinforcements, thus allowing the exploration of antennal gustatory discrimination via learning protocols of different nature. A study on gustatory discrimination in the fruit fly has paired proboscis extension to sweet tastants (CS) delivered to the tarsi with a noxious IR laser pulse (US) on the proboscis to generate conditioned inhibition^51^; yet, because the CS had an appetitive component (it elicits proboscis extension and reflects feeding motivation), it is hardly dissociable from a confounding reinforcing effect. In addition, as the response measured is appetitive, the study of pure aversive tastants was only accessible indirectly, via the mixing of the aversive tastant with sucrose (to elicit proboscis extension). This may generate the additional undesired effect of gustatory masking^51^ (see above). By contrast, gustatory SER conditioning excludes these effects by focusing on a robust defensive response, which is dissociated from feeding and allows stimulation with pure aversive tastants. While the antennae are not the only gustatory organs of the bee, similar experiments could be achieved at the level of the tarsi and the mouthpieces. The advantage of the antennae is not only that they are highly sensitive gustatory organs^13,48^, but also that they allow the spatial dissociation of gustatory stimuli and thus preclude the contamination of the same gustatory receptors by repeated stimulations with different stimuli. Delivering the same tastant repeatedly on the same antenna did not impede learning, thus discarding potential interferences due to the permanence of a tastant on gustatory receptors along the conditioning procedure. In such a case, the tastant would become a sort of unspecific contextual stimulus (i.e. a stimulus that is available before and after the electric shock) and would lose its capacity to predict the US. This was not the case in our experiments.

### Aminergic signaling underlying aversive gustatory learning

Our conditioning protocol allows access to the neural circuits underlying antennal gustatory learning. We showed that reinforcement pathways thought to mediate US signaling in the bee, such as the octopaminergic pathway in the case of sucrose reward^32–34^, mediate sweet-CS signaling in gustatory SER conditioning. Pharmacological blockade of octopaminergic signaling precluded learning only when sucrose was the punished conditioned stimulus. In *D. melanogaster*, sweet taste engages a distributed OA signal that reinforces memory through discrete subsets of dopaminergic neurons that convey the appetitive-reward information to the mushroom bodies^36,37^. The picture emerging from studies on appetitive reinforcement representation in the bee and the cricket brain is different as only octopaminergic signaling seems to be involved^32,33,64–66^. In our case, blockade of octopaminergic signaling was enough to suppress sucrose learning in an asymmetric way, consistent with the exclusive suppression of sugar signaling.

Blockade of dopaminergic signaling inhibited learning irrespective of the tastant used as CS. This effect is consistent with a suppression of shock signaling rather than gustatory signaling. It confirms the finding common to bees^35^, flies^67–69^ and crickets^65,66^ that DA signaling mediates aversive US (here the electric shock) signaling. Moreover, in the case of sucrose-shock learning, our results further indicate that octopaminergic and dopaminergic pathways have to converge in the bee brain to facilitate the learning of an association between a gustatory stimulus and shock. Anatomical characterizations of these pathways^70–73^ indicate that various regions of the bee brain such as the antennal lobes, the mushroom bodies and the subesophageal zone exhibit rich and coincident octopaminergic and dopaminergic innervation and are thus candidates for shock-induced sucrose plasticity. In the case of the fruit fly, the mushroom bodies have been found to play an important role for gustatory conditioning^51,74^ as calcium imaging upon gustatory stimulation revealed sparse, taste-specific and organ-specific activation in the Kenyon cell dendrites of the main calyx and the dorsal accessory calyx^74^. Also, the gamma lobes and a subset of dopaminergic input neurons are required for gustatory associative learning^74^. In the case of the honey bee, the specific sites of octopaminergic and dopaminergic interaction necessary for gustatory aversive learning remain to be determined.

## Material and Methods

### Insects

Honeybee workers, *Apis mellifera*, were obtained from colonies located in the apiary of the Research Center on Animal Cognition. The bees were brought to the laboratory and chilled on ice for 5 minutes until they stopped moving. They were then harnessed on individual holders (Fig. 1a) designed for aversive stimulation via delivery of an electric shock of 7.5 V^35,49,75^. Holders consisted of two brass plates fixed to a Plexiglas plate. Brass plates were connected to the output of the stimulator (50 Hz–AC current). Conductance gel was applied below the thorax and the abdomen to ensure efficient shock delivery. Low melting-point wax was used to immobilize the head and facilitate drug injection. Each fixed bee was fed with a droplet (5 µl) of 1 M sucrose solution and kept under resting for 1.5 h.

### Conditioning procedure

Each conditioning trial lasted 1 min. The bee was placed in the stimulation site in front of the air extractor and left for 20 s before being exposed to the tastant paired with the electric shock. Tastants were delivered to the antennae during 5 sec by means of a toothpick soaked in the tastant solution. The electric shock lasted 2 s; it started 3 s after onset of the gustatory stimulus and finished with it (Fig. 1b). The bee was then left in the setup for 35 s and then removed. The intertrial interval (ITI) was always 15 min. In order to keep this ITI, groups of 15 bees were trained one after the other. In this way, 15 min were required for the bees to complete each trial and to move to the next trial. An air extractor placed behind the conditioning apparatus prevented possible contamination by pheromone release. Several conditioning apparatuses were available to run several groups in parallel if necessary.

In *absolute conditioning* (Fig. 1b), the paired group received 5 conditioning trials that alternated with 5 blank trials^16^. During a blank trial, the bee was placed in the experimental position for 1 min and no specific stimulus was delivered. Blank trials were used in the paired group to equate the number of contextual experiences between paired and unpaired groups. In the unpaired group, trials lasted also 1 min, and only the tastant or the electric shock was presented. Thus, bees of both groups, paired and unpaired, were subjected to 5 gustatory stimulations, 5 electric shocks stimulations and 10 placements, so that only the explicit pairing of tastant and shock differed between groups. Sting extension responses (SER) to the gustatory stimuli were recorded for both groups. In addition, proboscis extension responses (PER) to sucrose were quantified whenever this stimulus was used as conditioned stimulus.

In *differential conditioning* (Fig. 1c), gustatory stimuli were presented in a pseudo-random sequence of 5 reinforced and 5 non-reinforced trials (e.g. ABBABAABABBA) starting with stimulus A or B in a balanced manner. Each gustatory stimulus (A, B) was delivered to a single antenna, left (L) or right (R), so that the experiment involved four groups of animals to achieve balance between antennal sides and reinforcement contingencies: A_L_+ vs. B_R_−, A_L_− vs. B_R_+ , A_R_+ vs. B_L_− and A_R_− vs. B_L_+ (Fig. 1d). We quantified the occurrence of SER to both tastants during conditioning trials.

Retention tests were performed 1 h after the last conditioning trial and consisted of presenting gustatory stimuli without punishment, using the same timing as in the conditioning trials. After differential conditioning, the sequence of tastant presentation (A, B) varied randomly from bee to bee. In all experiments, responses to the electric shock were measured before conditioning and after the retention test to verify the integrity of the unconditioned response. Only bees that consistently reacted to the electric shock were taken into consideration.

### Pharmacological drugs and injections

A small hole was pricked into the cornea of the median ocellus to allow the insertion of a 10 µl-syringe (World Precision Instrument), which was used to inject 200 nl of each drug solution^21^. Drugs were injected into the brain of immobilized bees along the median ocellar nerve^21^. This method ensures that drugs migrate through the ocellar tract and distribute within the bee brain in a fast (less than 5 min) and homogenous way^76^. Thirty min before the experiment, we injected: *epinastine hydrochloride*, an OA receptor antagonist^46^, *cis-(Z)-flupentixol dihydrochloride*, a DA receptor antagonist^47^; or PBS (control). Epinastine and flupentixol were obtained from Sigma-Aldrich (Saint-Quentin Fallavier, France). PBS was obtained from EUROMEDEX (Strasbourg, France). Injection time was chosen based on experiments, which showed that the effects of aminergic blockers reach a stable level approximately 30 min after drug application^35,77–79^. One mg of each drug was diluted in 1 ml PBS. Final concentrations obtained were 3.5 mM of epinastine and 1.94 mM of flupentixol. To test for dose-response effects, we prepared for each drug an additional dilution of 1:10000. In all cases, aliquots were kept in −20 °C until use. Each aliquot was used for one whole week and kept during this time in 4 °C.

### Data analysis

The occurrence of SER was recorded during the gustatory stimulation (CS) and during the electric stimulation (US). An observable sting extension was given a score of 1; incomplete sting movements were scored as 0. The percentage of bees responding to a conditioned tastant was then calculated. Two-way ANOVA (Statistica, StatSoft) was used for statistical comparisons. ANOVA procedures are applicable in the case of binary response variables despite their lack of normality if comparisons imply equal cell frequencies and at least 40 degrees of freedom of the error term^16,80,81^, conditions, which were met by our experiments. The use of repeated-measurement ANOVA allowed not only within-group analysis, but also between-group comparisons. Tukey tests were used for post hoc analyses. Retention performances were analyzed by means of a McNemar Test. Δ discrimination scores were compared against a zero value by means a one-sampled t test. A Wilcoxon test was used to compare Δ scores within groups. An alpha level of 0.05 was used throughout.

## Electronic supplementary material

Supplementary Information
