# Supplementary material for: Aversive gustatory learning and perception in honey bees
Source: Sci Rep. 2018 Jan 22;8:1343. doi: 10.1038/s41598-018-19715-1 (PMC5778057; doi:10.1038/s41598-018-19715-1)
Supplement: Supplementary file 1 — Supplementary Information [file 41598_2018_19715_MOESM1_ESM.pdf]

Supplementary Information

Aversive gustatory learning and perception in honey bees

Marie Guiraud[1,¥], Lucie Hotier[1], Martin Giurfa[1,*] and María Gabriela de Brito Sanchez[1,*]

*senior authorship shared

[1]: *Centre de Recherches sur la Cognition Animale (CRCA), Centre de Biologie Intégrative (CBI), Université de Toulouse; CNRS, UPS, 31062 Toulouse cedex 9, France*

[¥]: *Current address: Queen Mary University of London, School of Biological and Chemical Sciences, Biological and Experimental Psychology, Mile End Road, London E1 4NS, United Kingdom*

Figure S1

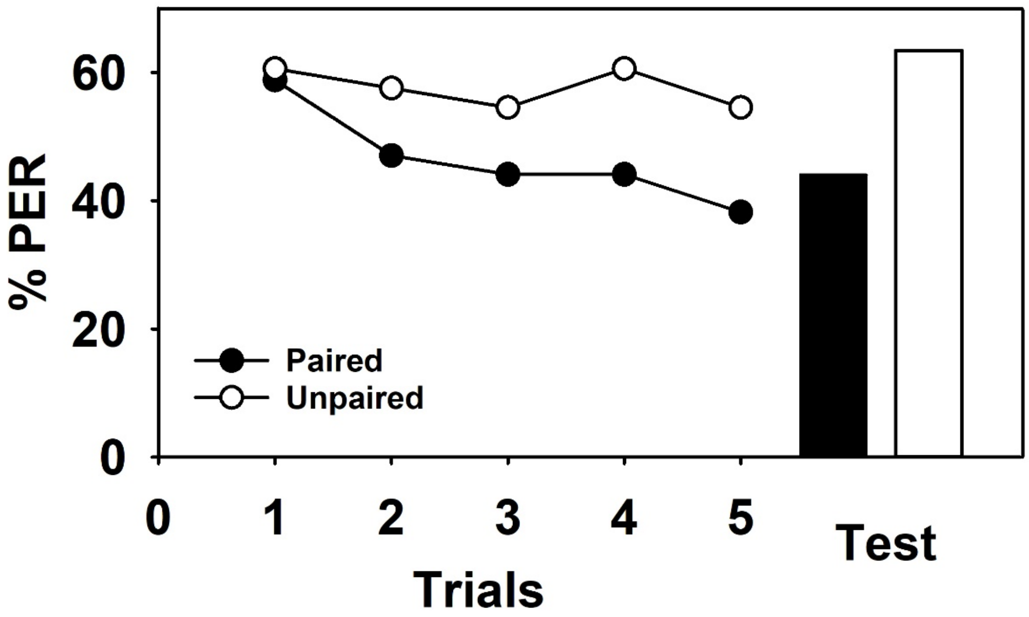

Figure S1: Appetitive responses (PER) to sucrose of bees from Fig. 2a. The responses of a paired group trained with sucrose solution contingent with electric shock (black circles; n = 34) and of an unpaired group presented with sucrose non-contingent with electric shock (white circles; n = 33) during 5 trials. PER to sucrose never reached typical levels observed in foragers trained appetitively (90-100%); it remained high (between 55% and 60%) and constant along trials in the unpaired group ($F_{4,128}$ = 0.15; P = 0.96). In the paired group, levels of PER (between 40% and 60%) were also lower than typical response levels and did not vary along trials ($F_{4,132}$ = 1.12; P = 0.35). No differences were observed between both groups ($F_{1,65}$ = 2.03; P = 0.16). One hour after conditioning, PER to sucrose was lower in the paired group (44%) compared to that of the unpaired group (64%) but the difference was again not significant ($F_{1,65}$ = 2.59; P = 0.11).

Figure S2

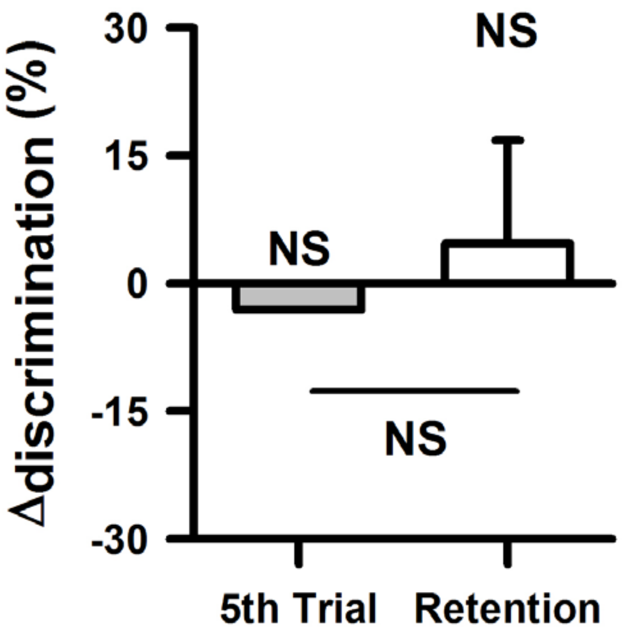

Figure S2: Control experiment showing that antennal side is not learned *per se* in differential gustatory SER conditioning. Bees (n = 64) were presented with a single tastant, sucrose solution 1.0 M, delivered alternately to the left and the right antenna, with only one antennal side being associated with shock. The $\Delta_{discrimination}$ indexes obtained for the last conditioning trial and for the retention test were not different from zero (t_{63} = 0.50; P = 0.62 and t_{63} = 0.77; P = 0.44, respectively) and did not differ from each other (t_{63} = 0.96; P = 0.34). Thus, bees were unable to learn the discrimination between sucrose punished and sucrose non-punished based on antennal side, showing that this variable was not learned during gustatory SER conditioning.
